# Curcumin Combination Chemotherapy: The Implication and Efficacy in Cancer

**DOI:** 10.3390/molecules24142527

**Published:** 2019-07-10

**Authors:** Bee Ling Tan, Mohd Esa Norhaizan

**Affiliations:** 1 Department of Nutrition and Dietetics, Faculty of Medicine and Health Sciences, Universiti Putra Malaysia, Serdang 43400, Selangor, Malaysia; 2 Laboratory of Molecular Biomedicine, Institute of Bioscience, Universiti Putra Malaysia, Serdang 43400, Selangor, Malaysia; 3 Research Centre of Excellent, Nutrition and Non-Communicable Diseases (NNCD), Faculty of Medicine and Health Sciences, Universiti Putra Malaysia, Serdang 43400, Selangor, Malaysia

**Keywords:** cancer, chemotherapy, curcumin, inflammation, nanoparticle, 5-fluorouracil

## Abstract

Many chemotherapeutic drugs have been used for the treatment of cancer, for instance, doxorubicin, irinotecan, 5-fluorouracil, cisplatin, and paclitaxel. However, the effectiveness of chemotherapy is limited in cancer therapy due to drug resistance, therapeutic selectivity, and undesirable side effects. The combination of therapies with natural compounds is likely to increase the effectiveness of drug treatment as well as reduce the adverse outcomes. Curcumin, a polyphenolic isolated from *Curcuma longa*, belongs to the rhizome of *Zingiberaceae* plants. Studies from in vitro and in vivo revealed that curcumin exerts many pharmacological activities with less toxic effects. The biological mechanisms underlying the anticancer activity of co-treatment curcumin and chemotherapy are complex and worth to discuss further. Therefore, this review aimed to address the molecular mechanisms of combined curcumin and chemotherapy in the treatment of cancer. The anticancer activity of combined nanoformulation of curcumin and chemotherapy was also discussed in this study. Taken together, a better understanding of the implication and underlying mechanisms of action of combined curcumin and chemotherapy may provide a useful approach to combat cancer diseases.

## 1. Introduction

Cancer is a major challenge worldwide, contributing to nearly 9.6 million deaths in 2018 [1]. Liver, stomach, colorectal, prostate, and lung cancers are the most common types of cancer among men; while thyroid, cervix, lung, colorectal, and breast cancers are the most common among women [1]. Despite tremendous improvements in treatment modalities have been made in recent decades, millions of cancer-associated deaths continue to escalate as a public health problem [2].

The most common therapeutic approaches for cancer treatment include immunotherapy and targeted therapy, chemotherapy, radiation, and surgery. Of these modalities, chemotherapy remains one of the most effective methods [3]. However, the efficacy and application of available anticancer chemotherapeutic drugs often fail to achieve complete cancer remission owing to the heterogeneity of cancer cells, show limited efficacy due to dose-limiting toxicity to the patients, and development of multidrug resistance [4]. Data from cell culture and animal models revealed that celecoxib, a specific cyclooxygenase-2 (COX-2) inhibitor, may suppress cancer cells such as colorectal cancer [5]. Nevertheless, the long-term use of celecoxib may increase the risk of cardiovascular toxicity [6]. Another chemotherapeutic drug, 5-fluorouracil (5-FU), has been used for the treatment of several cancers, including gastric [7], breast [8], liver [9], and prostate [10]. Notably, the study found that the anticancer efficacy of 5-FU was enhanced when its dosage was increased [11]. Unfortunately, the cytotoxicity of 5-FU was also increased in normal cells, and thus causes unacceptable toxicity to the patients [12]. Therefore, chemotherapy regimen that could enhance clinical outcomes is required for cancer patients. In order to overcome these issues, an ideal approach is to combine conventional chemotherapeutic with natural compounds to provide synergistic antitumor efficacy.

Natural products containing secondary metabolites have emerged as convincing candidate compounds for cancer treatment [13]. Curcumin, an active component with yellow phenolic pigment derived from dietary spice turmeric (*Curcuma longa*) rhizome, belongs to the *Zingiberaceae* plant family indigenous to south-eastern and southern tropical Asia [14]. In addition to its coloring, flavoring, and preservative properties in the diet, turmeric has been widely used for the treatment of many disorders and metabolic ailments such as certain cancer diseases, cough, skin wounds, and inflammation [15]. Emerging evidence has demonstrated that curcumin exerts many pharmacological activities, such as anti-inflammatory, antioxidant, and antitumor properties [16,17]. The safety of turmeric has been evaluated in several animal studies. Intriguingly, the data revealed that curcumin has advantages over traditional chemotherapeutic drugs, including broad anticancer activity and less toxic adverse outcome [18,19]. In a recent study, the data showed that pre-treatment with curcumin followed by 5-FU increased the susceptibility of the colon cancer cells/xenograft to the cytotoxicity of 5-FU [20]. Indeed, the molecular mechanisms underlying the anticancer activity of co-treatment curcumin and chemotherapy are complex and worth to discuss further. Therefore, this review aimed to address the molecular mechanisms of combined curcumin and chemotherapy in the treatment of cancer. The anticancer activity of combined nanoformulation of curcumin and chemotherapy was also discussed in this study.

## 2. Mechanisms of Action of Oxidative Stress-Induced Cancer 

Reactive oxygen species (ROS) are produced continuously in the body by mediating oxidative metabolism, immune activity, and mitochondrial bioenergetics [21]. However, when the ROS levels are elevated under stress, it may cause a detrimental impact on health [22]. The most frequent forms of ROS such as lipid peroxides, hydroxyl radical, hypochlorite, singlet oxygen, hydrogen peroxide, hyphochlorous acid, and superoxide anion are involved in the differentiation, death, growth, and progression of cells [22]. They can interact with proteins, membrane lipids, enzymes, and nucleic acids [23]. Short-term postprandial mitochondrial oxidative stress causes inflammation, which is predominantly mediated by nuclear factor-kappa B (NF-κB) [24].

Inflammation is an essential immune response to injury or infection in the body to regulate tissue homeostasis under certain circumstances such as swelling, redness, injury, infection, and irritation [25]. Among all chronic diseases, cancer is one of the prominent diseases contributed by chronic inflammation [26]. Inflammation is the seventh hallmark of cancer [27]. Both cancer and inflammation are linked through extrinsic and intrinsic pathways. For instance, oncogenes mediate the inflammatory microenvironments intrinsically and thus facilitate the progression and development of cancer extrinsically [28]. Indeed, the inflammation response is correlated to the tumor progression and the risk of malignancy [29]. Nearly 15% of cancer cases are activated through chronic inflammation and infection [30]. NF-κB is constitutively stimulated in many cancer diseases including pancreas, lung, liver, colon, and breast in response to carcinogens, for example, alcohol and tobacco [31].

The previous studies have demonstrated that transcription factors, for instance, NF-κB and signal transducer and activator of transcription 3 (STAT3), inflammatory enzymes including matrix metalloproteinase-9 (MMP-9) and cyclooxygenase-2 (COX-2), and inflammatory cytokines such as interleukins (IL)-1, -6, -8, and tumor necrosis factor alpha (TNF-α) are the key molecular mediators for inflammation-induced cancer cell proliferation, metastasis, angiogenesis, invasion, and inhibition of apoptosis [32]. Among these mediators, the transcriptional factor NF-κB is the main mediator of inflammation as it involves in the regulation of large arrays of cell adhesion molecules, cytokine receptors, and genes encoding cytokines [33]. The activity of NF-κB is triggered in response to proinflammatory cytokines and infectious agents by mediating IκB kinase (IKK) complex [34], suggesting a molecular link between cancer and inflammation [35]. NF-κB plays a pivotal role in the stimulation of some proinflammatory cytokines in several cell types such as epithelial cells, T cells, and macrophages [36]. Further, stimulation of NF-κB also triggers chemoresistance and radioresistance [7]. This finding suggests that NF-κB plays a crucial role in cancer and inflammation. Thus, anti-inflammatory agent targeting NF-κB and other associated products are potential in the treatment and prevention of cancers.

Given its tight association to NF-κB, IL-6 is an early candidate for the myeloid-derived factor that stimulates tumorigenesis [37]. As an example, patients with colon cancer demonstrated a high level of IL-6 [38]. Several studies reported by Bromberg and Wang [37] have also found the crucial role of IL-6 family of proinflammatory cytokines and their downstream effector STAT3 in colitis-associated colon cancer. Upregulation of STAT3 has been demonstrated in cancer patients, in which the STAT3 activity was positively linked to poor prognosis [39]. 

IL-6 is a predominant NF-κB-dependent cytokine that triggers the STAT3 activity. STAT3 is a cytoplasmic protein that serves as a transcriptional factor to trigger the inflammatory and immune response [32]. The stimulation of STAT3 involves a nuclear translocation, homodimerization, and tyrosine phosphorylation, where it interacts with DNA and modulates gene transcription [40,41]. Additionally, protein kinases, for example Janus-activated kinase (JAK) 3, 2, and 1 can induce the phosphorylation of STAT3 and subsequently trigger nuclear translocation [40].

An overexpression of TNF-α, the most potent proinflammatory cytokine, can stimulate cancer through activation of NF-κB expression [42]. In this regard, the blockage of TNF-α might have potential in the management and prevention of chronic disease such as cancer. Interleukins are another group of cytokines produced by macrophages. Several interleukins, for example IL-8, IL-6, and IL-1β play a vitally important role in the induction of proinflammatory response [43]. Aberrant expression of IL-1β, IL-6, IL-8, and TNF-α and activation of inducible nitric oxide synthase (iNOS) and COX-2 activity may contribute to oxidative stress, which in turn leads to inflammation [44]. Taken together, dysregulation of inflammation pathways appears to be involved in the pathogenesis of cancer.

## 3. Curcumin

Curcumin, a yellow pigment from *Curcuma longa*, is a major component of turmeric and is commonly used as a food-coloring agent and spice [45]. Curcumin is a low molecular weight polyphenol, is first isolated from turmeric in 1815, and the structure was delineated in 1910 as diferuloylmethane [46]. It is generally considered as the most active compound and contains about 2–8% of most turmeric preparations [47]. Curcumin is hydrophobic in nature and usually soluble in oil, ethanol, acetone, and dimethyl sulfoxide [48].

Curcumin has been recognized to protect biomembranes against peroxidative damage [49]. In general, lipid peroxidation is a free radical-mediated chain reaction that increased the damage of the cell membranes. Previous findings revealed that curcumin can inhibit the peroxidation through scavenging of reactive free radicals [50,51,52]. The ability of curcumin confers greater protection against oxidative damage has been attributed to their functional groups, such as phenyl rings, carbon-carbon double bonds, and β-diketone group [53].

## 4. Anticarcinogenic Potency and Molecular Mechanisms Induced by Curcumin

Growing evidence showed that curcumin and its analogs exert numerous pharmacological properties, for instance, antioxidant, anti-inflammation, and anticancer properties [45]. Of these properties, the anticancer activity of curcumin is known as one of the crucial effects of it. Many studies have demonstrated that curcumin (12 g/day for 3 months) induces apoptosis and antiproliferative against several types of cancer cell lines including prostate, breast, colorectal, pancreatic, and kidney [54,55]. Curcumin (0.1–3 mg/kg body weight) was shown to suppress telomerase reverse transcriptase enzyme [55] and reduce Bcl-2 expression [56]. Curcumin (2 µM) interacts with various proteins involved in angiogenesis, metastasis, and cell survival, as well as interferes with dysregulated signaling pathways in cancer cells, for instance phosphoinositide 3-kinase (PI3K)/Akt and NF-κB [57]. Curcumin exerts numerous effects by targeting several molecular and cellular pathways such as cell death, p53, Akt, mitogen-activating protein kinases (MAPK), microRNAs, and PTEN [45,58]. In this regard, these targets play a critical role in cancer pathogenesis and dysregulation of this pathway may lead to cancer progression and initiation [59]. The expression of NF-κB has been associated with inflammatory conditions as well as inducing a series of pathologic events involved in certain cancer [60]. NF-κB can be induced by endotoxin, ionizing radiation, carcinogen, free radical, and cytokine. Subsequently, these molecules trigger the activation of TNF that is linked to the upregulation of NF-κB expression [61]. Curcumin is well-recognized as an important regulator of NF-κB. For example, curcumin suppresses the activation of IKK that results in the translocation of NF-κB to the nucleus [62]. Furthermore, data from in vitro and in vivo studies have also shown a potent cytotoxic effect of curcumin on several pancreatic cancer cells through the inhibition of oxidative stress and angiogenesis as well as via the induction of apoptosis [63]. Dose-escalating study has demonstrated the safety of curcumin at doses up to 12 g/day over 3 months [64].

In addition to the targets mentioned above, microRNAs (miRNAs) are known as small non-coding RNA that plays a crucial role in various physiological conditions, including differentiation, growth, angiogenesis, and apoptosis [65,66]. Dysregulation of these molecules can upregulate and downregulate several cellular and molecular targets that lead to the progression of cancer [67]. Compelling evidence suggests that curcumin exerts its anticancer properties by targeting different miRNA expression such as miR-181b, miR-203, miR-9, miR-19, miR-21, miR203, miR-9, and miR-208 expression [58,68,69]. A study reported by Jin et al. [70] evaluated the curcumin (5-40 µM) in relation to miRNA levels. The data showed that curcumin (10 and 20 µM) upregulates miR-192-5p by modulating the PI3K/Akt signaling pathway in non-small cell lung cancer cells. The data reported by Schwertheim et al. [71] further demonstrated that curcumin (50 µM) upregulates miR-21 expression in thyroid carcinoma. Notably, phase I clinical trial for Bowen’s disease (squamous cell carcinoma in situ) conducted by Cheng et al. [72] showed that no treatment-associated toxicity up to 8000 mg/day for 3 months. The histological analysis further revealed that the precancerous lesions were reduced by 33% in patients with Bowen’s disease [72]. Despite extensive studies have shown that curcumin induces cytotoxicity against cancer cells by targeting different mechanisms, curcumin combination chemotherapy is more likely to enhance the synergistic effect of cancer cells to treatment. Data from in vitro study showed that curcumin (20 µM) sensitizes human gastric cancer cells to 5-FU (100 µM) via suppression of NF-κB signaling pathway [73]. In this regard, this can reduce the concentrations of chemotherapeutic drug and decrease the adverse effects of drugs [56].

## 5. The Synergistic Effect of Curcumin Combination Chemotherapy

### 5.1. In Vitro and In Vivo 

Emerging preclinical evidence indicates that combination therapies promote anticancer efficacy without elevating toxicity [74]. Docetaxel (30 or 75 mg) has been clinically approved and widely used for the treatment of metastatic castration-resistant prostate cancer [75]. However, prolonged treatment with docetaxel could cause severe toxicity in patients [76]. A study by Banerjee et al. [77] found that the combined treatment of docetaxel (10 nM) and curcumin (20 µM) for 48 h significantly inhibited the proliferation and induced apoptosis in prostate cancer (PC-3) (DU145 and PC3) cells compared to the curcumin and docetaxel alone. The data further demonstrated that curcumin enhances the efficacy of docetaxel in PC-3 cells via modulation of COX-2, p53, NF-κB, phospho-Akt, PI3K, and receptor tyrosine kinase (RTK) [77]. In this regard, this finding implies that combining curcumin with conventional chemotherapy may act as an effective treatment regimen for patients with prostate cancer to reduce cytotoxicity and overcome drug-resistant induced by docetaxel.

Metformin (1500–3000 mg/day for 6 months) is recognized as a well-tolerated anti-diabetic drug [78]. Importantly, several studies revealed that metformin decreases the risk of many cancers including hepatocellular carcinoma and prostate cancer [79,80]. Clinical evaluation of metformin (500 mg daily for 1 week followed by 1 g twice daily for a week) for antineoplastic and chemopreventive effects has bypassed the phase I assessment and directly entered to phase II/III trials in several cancers due to less toxicity records in diabetic patients [81]. Data from an in vitro study revealed that combined metformin (10 mM) and curcumin (5 and 10 µM) can induce apoptosis and inhibit metastasis and invasion in HepG2 and PLC/PRF/5 cells. The anticancer effects could be attributed to the vascular endothelial growth factor (VEGF), MMP2/9, and vascular endothelial growth factor receptor 2 (VEGFR-2) inhibition, PTEN and p53 activation, and epidermal growth factor receptor (EGFR)/STAT3 and NF-κB/mTOR/Akt/PI3K suppression [82]. Data from an in vivo study further showed that co-treatment with metformin and curcumin significantly suppressed hepatocellular carcinoma compared to curcumin (60 mg/kg) and metformin (150 mg/kg) alone in a xenograft mouse model [82]. 

5-FU alone (10 μM) or in combination with other chemotherapeutic drugs has been widely applied for the treatment of colorectal cancer [83,84]. However, multidrug resistance was often developed in patients with colorectal cancer administered with 5-FU-based regimen [85]. The previous findings suggest that the combination of 5-FU and curcumin may overcome the drug-resistant induced by 5-FU. Pre-treatment with curcumin (5 µM) enhanced the chemosensitization of 5-FU (0.1 µM) and reversed the multidrug resistance in resistant mismatch repair (MMR)-deficient human colon cancer cells compared to 5-FU alone [86]. Combination of curcumin (10 μM) and 5-FU (0.1 mM)/oxaliplatin (5 μM) enhanced the synergistic antitumor activity in gastric cancer (BGC-823) cell lines compared to curcumin or 5-FU/oxaliplatin alone by downregulating the Bcl-2 mRNA and protein expression and activating the Bax and caspases-3, 8, and 9 expressions [87]. The study further demonstrated that the combination of curcumin (10 mg/kg) and 5-FU (33 mg/kg)/oxaliplatin (10 mg/kg) show potent growth inhibition of BGC-823 xenograft tumors compared to folinic acid, 5-FU, oxaliplatin (FOLFOX) or curcumin alone [87]. In addition, a combination of curcumin (50 mg/kg/day for 40 days) and 5-FU (20 mg/kg once every 2 days for 40 days) also inhibits the cell proliferation against 5-FU resistant cells via suppression of epithelial-to-mesenchymal transition (EMT) compared to 5FU alone [88]. In the context of breast cancer, a combination of curcumin (10 µM) and 5-FU (10 µM) significantly inhibited the cell viability and enhanced apoptosis compared to 5-FU alone in vitro [89]. In addition to the effects mentioned above, a study reported by Yang et al. [90] has shown that combination of 5-FU (50 µmol/L) and curcumin (25 µmol/L) can enhance the cytotoxicity against human gastric cancer (AGS) cells compared to 5-FU or curcumin alone. In a further study focused on inflammation outcomes, Yang et al. [90] found that the protein expression of COX-2 and NF-κB in human gastric cancer (MKN45) cells were diminished after co-treatment with 5-FU (50 µmol/L) and curcumin (25 µmol/L). This finding implies that curcumin sensitizes gastric cancer cells to 5-FU by modulating inflammatory molecules. The anti-gastric cancer activity is not only shown in in vitro study, data from an animal study further demonstrated that curcumin enhanced the anticancer activity of 5-FU (52 mg/kg 5-FU + curcumin 74 mg/kg, every 3 days for 6 times in total) compared to 5-FU or curcumin alone, and without increasing the toxicity in nude mice bearing MKN45 tumor xenografts [90].

Doxorubicin, one of the active single-agent drugs, is widely used for the treatment of cancers, including leukemia, lung, brain, prostate, ovarian, and breast. However, the clinical use of doxorubicin often led to critical cardiotoxicity and developed multidrug resistance [91]. Substantial evidence revealed that curcumin (4 mg/kg every 2 days for a total of 7 injections) exhibits a better treatment efficacy of doxorubicin (0.4 mg/kg) in cancer due to its efflux inhibitory effect of curcumin [92,93,94]. A study conducted by Guorgui et al. [95] has shown that combination of curcumin (5 µM) and doxorubicin (0.4 mg/mL) demonstrated a stronger additive effect by reducing the proliferation of Hodgkin lymphoma (L-540) cells by 79%. The pharmacokinetic study also revealed that curcumin (5 mg/kg) could enhance the absorption of doxorubicin (5 mg/kg) and decrease drug efflux in vivo, suggesting that curcumin downregulates the intracellular levels of ATP-binding cassette (ABC) drug transporters [96].

Cisplatin-based combination therapy has emerged as a standard therapy for metastatic and advanced bladder cancer [97], demonstrating 15–20% improved survival and 50–70% response rate. However, nearly 30% of patients do not respond to initial chemotherapy and show recurrence within 1 year [98]. Cisplatin is an inorganic platinum agent which can induce DNA-protein and interstrand and intrastrand DNA crosslinks [99]. Despite this crosslink can induce apoptosis and inhibit cell proliferation [100], the efficacy of cisplatin is limited by the development of cell resistance. Co-treatment with curcumin (10 µM) and cisplatin (10 µM) has shown a potent synergistic effect by activating caspase-3 and upregulating phospho-mitogen-activated protein kinase (p-MEK) and phospho-extracellular signal-regulated kinase 1/2 (p-ERK1/2) signaling in bladder cancer cell lines (253J-Bv and T24) compared to curcumin or cisplatin alone [101]. In addition to the effects observed on bladder cancer, the combination of curcumin and cisplatin was shown to upregulate the expression of miR-186 via modulation of Twist1 in ovarian cancer compared to cisplatin alone [102].

**Figure 1:**
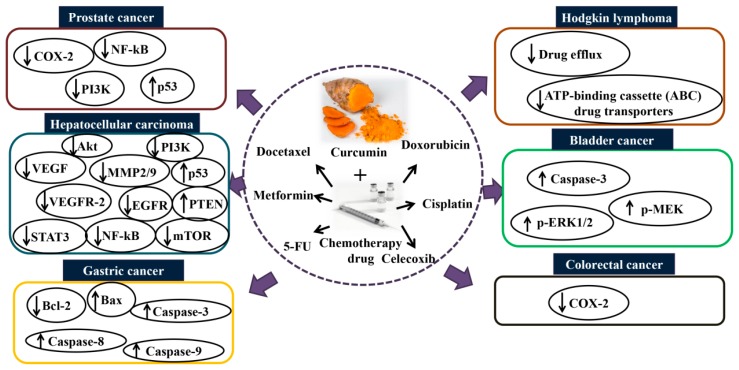
Mechanisms of action of combination curcumin and chemotherapy drugs in vitro and in vivo. Co-treatment with curcumin and chemotherapy drugs such as docetaxel, metformin, 5-fluorouracil, doxorubicin, cisplatin, and celecoxib enhance the synergistic effect via modulating several signaling pathways and thus inhibit cancers such as prostate, hepatocellular, gastric, Hodgkin lymphoma, bladder, and colorectal. Akt: protein kinase B; COX-2: cyclooxygenase-2; EGFR: epidermal growth factor receptor; MMP2/9: matrix metalloproteinase-2/9; mTOR: mammalian target of rapamycin; NF-κB: nuclear factor kappa B; p-ERK1/2: phospho-extracellular signal-regulated kinase 1/2; PI3K: phosphoinositide 3-kinase; p-MEK: phospho-mitogen-activated protein kinase; STAT3: signal transducer and activator of transcription 3; VEGF: vascular endothelial growth factor; VEGFR2: vascular endothelial growth factor receptor 2.

Besides the effects mentioned above, celecoxib is another selective inhibitor of COX-2, an enzyme induced by different stimuli including inflammation [103]. Celecoxib (75 µM for 16 h) has shown an ability to induce apoptosis and suppress tumor angiogenesis in several types of cancer [103]. However, the long-term use of celecoxib leads to an adverse outcome such as cardiovascular toxicity [104]. Combination of curcumin and celecoxib was shown to reduce cancer cell growth in vitro compared to celecoxib alone. A study reported by Lev-Ari et al. [105] revealed that curcumin (10–15 µmol/L) and physiological dosage of celecoxib (5 µmol/L) exhibited a synergistic inhibitory effect against human colorectal cancer (HT-29) cells. The study showed that the combination of curcumin and celecoxib induces apoptosis in HT-29 cells via downregulation of COX-2 expression, suggesting that curcumin synergistically augments the growth inhibitory effects of celecoxib in human colon cancer cell lines in vitro. Collectively, the synergistic effects of curcumin and chemotherapy on cancer are important and worth attention, particularly in patients receiving antineoplastic or anti-inflammatory drugs. Figure 1 summarizes the mechanisms of action of combination curcumin and chemotherapeutic drugs in vitro and in vivo.

### 5.2. Clinical Trials 

In addition to the effects observed in both in vitro and in vivo models, the implication of curcumin combination chemotherapy has been reported in several clinical trials (Table 1). The activity and feasibility of gemcitabine and curcumin were assessed in patients with pancreatic cancer. Previous studies suggest that utilization of curcumin at different dosage is safe in animal and human models [106,107]. Indeed, several studies reported by Waghela et al. [106] and Shankar et al. [107] have demonstrated the concentration of curcumin could be tolerated even at very high doses. However, low doses of curcumin are related to the therapeutic effects of several cancers [108].

A phase-I escalated clinical trial evaluated the feasibility and tolerability of the docetaxel and curcumin in 14 patients with advanced or metastatic breast cancer. Curcumin was orally given at a dosage of 500 mg/day for seven days and increased until dose-limiting toxicity. The data revealed that administration of docetaxel (100 mg/m^2^) and curcumin (500 mg/day) show promising biologic response in the chemoprevention by reducing carcinoembryonic antigen (CEA) tumor marker [109]. The maximum tolerated dose for curcumin is 6000 mg/day for seven consecutive days every three weeks in combination with a standard dosage of docetaxel [109]. In a study reported by Kanai et al. [112] focusing on the safety of combination therapy using 8 g oral curcumin daily with gemcitabine-based chemotherapy (1000 mg/m^2^ on day 1 and 8) and (60 mg/m^2^ of S-1 orally for 14 consecutive days every 3 weeks) in 21 patients with gemcitabine-resistant pancreatic cancer, the patients were shown no dose-limiting toxicities in the phase I study, suggesting that oral curcumin 8 g/day is safe and feasible in patients with pancreatic cancer. In a prospective phase II trial evaluated the safety and efficacy of curcumin (2000 mg/die continuously (4 capsules, each of 500 mg, every day) and gemcitabine (10 mg/m^2^) on 44 advanced and metastatic pancreatic cancer patients, the data showed that the median progression-free survival and overall survival were 8.4 and 10.2 months, respectively. This finding implies that complementary therapy to gemcitabine with phytosome complex of curcumin is safe and efficient to translate in a good response rate in first-line therapy of advanced pancreatic cancer [114]. Moreover, curcumin (5 µM) was found to enhance FOLFOX-based chemotherapy (2 µM oxaliplatin + 5 µM 5-FU) in patient-derived colorectal liver metastases cultures [113]. The phase I escalation study demonstrated that curcumin is safe and tolerable in combination with FOLFOX chemotherapy in patients with colorectal liver metastases at a dosage up to 2 g daily [113]. There are several limitations for drawing conclusions from the clinical trials include (1) small sample size; (2) different daily dosage of curcumin; (3) short duration of treatment; (4) other factors such as age may confound the results; and (5) possible bias from the interpreting pathologist. Additionally, assessment of dose-response relationship and cancer has not been evaluated. It would be useful to have moderate and high curcumin combination chemotherapy compared in the same study. Based on the evidence, the synergistic role played by curcumin and chemotherapeutic drugs worth study in-depth in a large clinical trial.

## 6. Nanoformulation of Curcumin Combination Chemotherapy

Despite curcumin pharmacological properties were reported in vivo and in vitro models, poor aqueous solubility and low stability may limit the clinical usefulness of curcumin combination chemotherapy. These unfavorable effects have hampered the quantity of curcumin absorbed, and thus severely limit its bioavailability. Nanoformulation-based combination therapy has emerged as a potent approach for drug delivery system [115]. It has received attention as it can resolve problems associated with conventional therapeutic agents by improving intracellular drug concentrations and enhancing the synergistic activity for cancer therapy [116]. Thus, the application of nanotechnology could improve the efficacy and enhance bioavailability by increasing permeation in the small intestine, preventing degradation in the intestinal environment, increasing plasma half-life, and enhancing the efficacy [117].

Some attempts have been made to improve the bioavailability of curcumin. Formulation of curcumin with d-α-tocopheryl polyethylene glycol 1000 succinate-stabilized curcumin (TPGS-curc) has been widely studied [118,119,120]. The data revealed that TPGS-curc (10 mg/kg body weight) shows a better in vivo kinetic profile than curcumin alone, suggesting that TPGS an ideal formulation to improve curcumin bioavailability in vivo. In addition, formulating curcumin in solid lipid nanoparticles (SLN-curc) (100 mg/kg of curcumin, 5 days per week for 18 days) exhibited a strong anticancer activity compared to curcumin alone (100 mg/kg, 5 days per week for 18 days) [95]. The data showed that SLN-curc increased cell cycle regulator (p21) and diminished the anti-apoptotic (XIAP and Mcl-1) levels [95]. In support of the efficacy of nanoformulation-based curcumin, in vitro study has demonstrated that the efficacy of cell proliferation in dendrosomal curcumin (8.31-13.45 µM) is superior compared to curcumin alone (13 to 30 µM) [120]. The data further showed that dendrosomal curcumin therapy (13.45 and 11.66 µM) significantly increased the GAS5 and TUSC7 levels, a tumor suppressor gene expression, in human breast cancer (MCF-7, SKBR3, and MDA-MB231) cell lines in a dose-dependent manner [120]. In this regard, this finding implies that the overexpression of GAS5 might reduce chemotherapy resistance. This finding is in line with the previous study reported in in vitro model for bladder cancer cells, in which the overexpression of GAS5 enhances bladder cancer cells sensitivity to doxorubicin [121]. In the context of a clinical trial, a phase I study evaluated the safety and tolerability of increasing dose of liposomal curcumin (100-300 mg/m^2^) in patients with metastatic or advanced cancer [122]. The data showed that 300 mg/m^2^ liposomal curcumin over 6 h is the maximum tolerated dose in these heavily pre-treated patients, suggesting the recommended starting dose for anticancer trials [122].

Nanodrug delivery system has been found to be an efficient approach to enhance the synergistic effect (Table 2 and Table 3). Co-delivery micelles of doxorubicin and curcumin (8 µg/mL doxorubicin and 8 µg/mL curcumin) significantly reversed multidrug resistance compared to doxorubicin (8 μg/mL) by targeting CD44. The data from an animal study revealed that co-delivery micelles of doxorubicin and curcumin inhibited the growth of tumor in 4T1 tumor-bearing mice [123]. Polymeric micelles, a core-shell structure with hydrophilic shell and hydrophobic core of micelles, can effectively accumulate in the tumor by enhancing the permeability and retention, and subsequently increasing the therapeutic effects of chemotherapeutic drugs [124]. A study by Xiao et al. [125] further supported the role of nanodrug co-delivery system in the modulation of cancer cell sensitivity. Combined delivery of chitosan-functionalized camptothecin/curcumin-loaded poly(lactic acid/glycolic acid) polymeric nanoparticle (6 mg) can enhance the synergistic effects against colon-26 cells compared to camptothecin or curcumin alone [125]. In another study, Nguyen et al. [126] evaluated a combined nanodrug delivery system by incorporating a chemotherapeutic agent, methotrexate (MTX) (2.5 mg/kg), and a photosensitizer material (polyaniline) (5 mg/kg) into hybrid polymer nanoparticles. The data from both in vitro and in vivo studies showed that chemo-phototherapy enhanced the synergistic effect and inhibited somatostatin-positive cancer compared to MTX or polyaniline nanoparticle alone [126]. MTX, an antimetabolite drug, is one of the effective therapeutic agents for disease associated with aberrant cell growth [127]. Dendritic chitosan grafted methoxy polyethylene glycol (mPEG) coated with magnetic nanoparticle is another magnetic nanocarrier that used for co-delivery doxorubicin and MTX. Data from in vitro study demonstrated that combination drug delivery could enhance the synergistic effects and thereby better alleviate the adverse outcomes compared to doxorubicin or MTX alone [128]. Taken together, this newly developed nanoformulation by encapsulating curcumin and chemotherapeutic drugs hold a great promising as a future therapy for cancer.

## 7. Conclusions 

Chemotherapeutic drugs such as doxorubicin, irinotecan, 5-fluorouracil, cisplatin, and paclitaxel have been applied in cancer therapy. Nonetheless, the efficacy of chemotherapy is limited due to the development of drug resistance and undesirable outcomes. The combination of therapies with natural antitumor compounds was shown to enhance the effectiveness of drug treatment and decrease the toxic effect. Curcumin has found to be cytotoxic and exert chemopreventive activities in nature to a wide variety of cancer cell lines and animal tumor models via multiple molecular mechanisms targeting all stages of carcinogenesis. However, water insolubility and low stability of curcumin have hampered the quantity of curcumin absorbed and its bioavailability. Nanoencapsulation has emerged as a potent strategy to enhance the therapeutic potential of conventional drugs. Co-delivery of nanoparticle-based curcumin and chemotherapy has shown efficacy for improving intracellular drug concentration and enhancing the synergistic effect in cancer therapy by sensitizing cancer cells towards chemotherapeutic drugs. This concurrent administration enhances anticancer efficacy and reduces the use of chemotherapy drugs. Subsequently, this can reduce the adverse outcome caused by drugs. Although in vitro and in vivo studies have demonstrated the synergistic effects in co-delivery of nanoformulation of curcumin and chemotherapeutic drugs, further studies are warranted to elucidate the benefit-risk profile of curcumin as well as concurrent administration of nanoformulation of curcumin and drugs in a large clinical trial. Taken together, this evidence may pave the way for a useful approach to combat cancers, providing that this strategy involves a regimen with a low dosage of side effects.

## Figures and Tables

**Table 1:** Clinical trials conducted in a combination of curcumin and chemotherapy.

Types of Cancer	Treatment	Participants	Findings	References
Advanced or metastatic breast cancer	Curcumin (500 mg/day) and escalated until a dose-limiting toxicity + docetaxel (100 mg/m^2^) for 7 days every 3 weeks	14 patients	Improves biological and clinical responses	[109]
Pancreatic cancer	Curcumin (8000 mg/day) + gemcitabine (1000 mg/m^2^) weekly	17 patients	Time to tumor progression was 1-12 months and overall survival was 1-24 months	[110]
Chronic myeloid leukemia	Imatinib (400 mg twice a day for 6 weeks) Group B [Turmeric powder (5 g three times/day) + imatinib (400 mg twice a day)] for 6 weeks	50 patients	The suppressive effect of nitric oxide levels was noted at Group B	[111]
Pancreatic cancer	Curcumin (1000 mg/day) + gemcitabine (1000 mg/m^2^ on day 1 and 8) and 60 mg/m^2^ of S-1 orally for 14 consecutive days every 3 weeks	21 patients	Median survival time after initiation of curcumin was 161 days and 1-year survival rate was 19%	[112]
Colorectal liver metastases	5 µM curcumin + 2 µM oxaliplatin + 5 µM 5-FU	12 patients	Curcumin enhanced the FOLFOX-based chemotherapy	[113]
Pancreatic cancer	Curcumin (2000 mg/die continuously (4 capsules, each of 500 mg, every day) + gemcitabine (10 mg/m^2^)	44 patients (13 locally advanced and 31 metastatic)	Median progression-free survival and overall survival were 8.4 and 10.2 months, respectively	[114]

**Table 2:** Summary of nanoformulation curcumin and chemotherapeutic drugs in vitro.

Types of Cancer	Co-Delivery System	Treatment	Cancer Cell Lines	Findings	References
Colon cancer	Cationic polymeric nanoparticles	Camptothecin and curcumin	Colon-26 cells	Enhances synergistic effects of anticancer activity	[125]
Liver cancer	Lipid nanoparticle	Curcumin and doxorubicin	HepG2 cells	Enhance cytotoxicity and decrease inhibitory concentration in HepG2 cells	[129]
Breast cancer	Liposomal	Paclitaxel and curcumin	MCF-7 cell lines	Effectively kills the cancer cells compared to individual treatment	[130]
Breast cancer	Dendritic chitosan grafted methoxy polyethylene glycol (mPEG) coated magnetic nanoparticles	Doxorubicin and methotrexate	MCF7 cell lines	Enhances synergistic effects and alleviates adverse outcome	[128]
Breast cancer	Transferrin-decorated nanoparticles	Curcumin and doxorubicin	MCF-7 cells	Combination of transferrin-poly(ethylene glycol)-curcumin (Tf-PEG-CUR) and doxorubicin nanoparticle exhibited higher cytotoxicity in MCF-7 cells compared with Tf-PEG-CUR nanoparticle alone	[131]
Colon cancer	PEGylated long-circulating liposomes	Curcumin and doxorubicin	C26 murine colon cancer cells	Exerts strong antiproliferative effects via inhibition of the angiogenic/inflammatory proteins such as TNF-α, TIMP-2, and IL-6	[132]
Breast cancer	Albumin nanoparticles	Curcumin and doxorubicin	MCF-7 resistant breast cancer cells	Shows lower viability than the cells treated with a combination of curcumin and albumin nanoparticle or combination of doxorubicin and albumin nanoparticle	[133]
Liver cancer	Biotin-/lactobionic acid–modified poly(ethylene glycol)-poly(lactic-co-glycolic acid)-poly(ethylene glycol) (BLPP) copolymer	Curcumin and 5-FU	HepG2 cells	Exhibits higher cellular uptake, strong cytotoxicity for tumor cells	[134]
Pancreatic cancer	Iron oxide nanoparticles	Curcumin and gemcitabine	Human pancreatic cancer (HPAF-II and Panc-1) cell lines	Effectively delivers bioactive curcumin to pancreatic cells, simultaneously enhances gemcitabine uptake	[135]

BLPP: biotin-/lactobionic acid–modified poly(ethylene glycol)-poly(lactic-co-glycolic acid)-poly(ethylene glycol); IL–6: interleukin-6; TIMP-2: tissue inhibitor of metalloproteinases 2; TNF-α: tumor necrosis factor α; 5-FU: 5-fluorouracil.

**Table 3:** Summary of nanoformulation curcumin and chemotherapeutic drugs in vivo.

Types of Cancer	Co-Delivery System	Treatment	Animal Model	Findings	References
Lung cancer	Methoxy poly(ethylene glycol)-poly(caprolactone) (MPEG-PCL) micelles	Curcumin and doxorubicin (5 mg/kg) intravenous tail injection every 5 days until the control mice became weak	The female C57 mice (aged 6-8 weeks) (n = 40) mice were injected subcutaneously with 100 µL of LL/2 cell suspension (1 × 10^6^) into the right flank. The tumor-bearing mice were randomly divided when the mean tumor diameter reached about 6 mm	1. Tumors in the groups treated with curcumin and doxorubicin/MPEG-PCL were smaller than those receiving the other treatments (*p* < 0.05) 2. Inhibited the growth of subcutaneous LL/2 lung carcinoma (*p* < 0.05) 3. Induced apoptosis of tumor tissue and inhibited tumor angiogenesis, as shown in TUNEL assay and CD31 staining (*p* < 0.05)	[93]
Liver cancer	Lipid nanoparticle	Curcumin and doxorubicin (2 mg/kg doxorubicin) for 20 weeks, intravenously injected once a week	Diethylnitrosamine-induced hepatocellular carcinoma mice (n = 32) 24 mice were administrated by oral administration of diethylnitrosamine solution in sesame oil (0.1 g/mL) at 40 mg/kg once a week for 15 weeks. 8 mice were administrated with sesame oil only (normal mice)	1. The liver/body weight (*p* < 0.05) and serum ALT and AST levels (*p* < 0.01) were significantly decreased in curcumin and doxorubicin-lipid nanoparticle group 2. The mRNA and protein levels of Bax/Bcl-2 (*p* < 0.01) were all increased in tumor tissue from curcumin and doxorubicin-lipid nanoparticle group compared to the doxorubicin-lipid nanoparticle group 3. The immunohistochemistry analysis induced expression of caspase-3 4. The expression of c-myc and PCNA decreased significantly in curcumin and doxorubicin-lipid nanoparticle (*p* < 0.01) compared to the control group 5. The mRNA and protein levels of VEGF in curcumin and doxorubicin-lipid nanoparticle were significantly decreased compared to the control group (*p* < 0.05) 6. The mRNA levels of *MDR1* and *Bcl-2*, as well as the protein levels of P-gp and Bcl-2 were all decreased in curcumin and doxorubicin-lipid nanoparticle group compared to the doxorubicin-lipid nanoparticle group (*p* *<* 0.01)	[136]
Breast cancer	Polymeric micelles	Curcumin (10 mg/kg) and doxorubicin (10 mg/kg) for 12 days	The female BALB/c mice (n = 60) were injected with 1 × 10^6^ 4T1 cells into the right axilla of mice. When the tumor reached about 100 mm^3^, 4T1 tumor-bearing mice were randomly divided into 6 groups (n = 10)	1. Curcumin and doxorubicin polymeric micelles treated group exhibited a considerable tumor inhibition compared to the saline-treated group (*p* < 0.001) 2. The AST, LDH, CK, and CKMB were significantly reduced compared to the mice treated with doxorubicin polymeric micelles (*p* < 0.05) 3. No pathological damages were found in heart, liver, spleen, lung, and kidney in the mice treated with curcumin and doxorubicin polymeric micelles by using H&E staining 4. Tumors treated with curcumin and doxorubicin polymeric micelles had enhanced dark brown spots by using TUNEL assay, indicating that drug encapsulated in micelles could enhance tumor cell apoptosis and showed better antitumor effects	[123]
Breast cancer	Transferrin-poly(ethylene glycol)	Curcumin (50 mg/kg) and doxorubicin (50 mg/kg) were injected into the mice by tail vein for 7 weeks	BALB/c mice were inoculated subcutaneously with 1 × 10^6^ MCF-7 cells. MCF-7 tumor xenografts were grown in BALB/c mice and estrogen was provided as a β-estradiol pellet 1 week prior to the injection of the cells. The tumors were allowed to develop on the posterolateral side of the mice for 1 week prior to treatment to obtain the breast cancer-bearing animal model. The mice were randomly divided into 6 groups	Compared with curcumin and doxorubicin, transferrin-poly(ethylene glycol)-curcumin/doxorubicin nanoparticles presented a remarkably higher inhibition effect towards tumor growth (*p* < 0.05)	[131]
Liver cancer	Biotin-/lactobionic acid–modified poly(ethylene glycol)-poly(lactic-co-glycolic acid)-poly(ethylene glycol) (BLPP) copolymer	Curcumin (10 mg/kg) and 5-FU (4 mg/kg). The mice were injected once at an interval of 2 days of a total of 4 injections through the tail vein for 30 days	The BALB/c nude mice (n = 18) were inoculated subcutaneously with HepG2 cells (2 × 10^6^). The mice were randomly divided into 6 groups (n = 3) when the tumor volume reached about 50 mm^3^	1. The tumors in BLPP/curcumin +5-FU nanoparticle were approximately 8 times smaller than the tumor volume observed in the control (phosphate-buffered saline) group (*p* < 0.001) 2. The mice treated with BLPP/curcumin +5-FU nanoparticles induced tumor apoptosis or necrosis significantly compared to the BLPP/curcumin nanoparticle (*p* < 0.05) 3. The western blotting analysis showed that BLPP/curcumin + 5-FU nanoparticle significantly decreases the DPYD expression compared to the BLPP/5-FU nanoparticle and the control groups (*p* < 0.05) 4. p53 protein expression was higher in BLPP/curcumin groups than in BLPP/5-FU nanoparticle group (*p* < 0.05) 5. Bcl-2 protein expression of BLPP/curcumin + 5-FU was lower than that of the BLPP/5-FU nanoparticle, BLPP/curcumin nanoparticle, and control groups; while the expression of cytochrome c was higher (*p* < 0.05)	[134]
Pancreatic cancer	Superparamagnetic iron oxide nanoparticle (SPION) formulation of curcumin (SP-CUR)	2 treatments groups [Curcumin (100 µg dissolved in 100 μL of 0.1% Tween 20) and gemcitabine (300 μg dissolved in 50 μL of phosphate-buffered saline)] and another two groups were treated with an intraperitoneal injection of 100 μg curcumin loaded SP-CUR and combination with gemcitabine, respectively. Treatments were administered twice weekly for 7 weeks	HPAF-II cells (1.0 × 10^6^) and human pancreatic stromal cells (stromal component; 0.5 × 10^6^) were suspended in 50 μL of HBSS media containing 1% (*v*/*v*) matrigel and injected into the parenchyma of the pancreas in old male athymic nude mice (6 weeks old). Five days later, mice were randomly divided into five groups (n = 8)	1. The bioluminescence imaging results showed a significant (*p* < 0.05) decrease in the pancreatic tumor volume of SP-CUR + gemcitabine-treated mice compared to the vehicle-treated mice 2. SP-CUR + gemcitabine-treated mice showed a significant decrease in the tumor weight of pancreas compared to the gemcitabine alone (*p* < 0.0001) 3. None of the mice treated with SP-CUR + gemcitabine were recorded for distant metastasis 4. Immunoblotting and immunohistochemistry analyses showed that SP-CUR + gemcitabine inhibited SHH, NF-қB, Gli-1 and Gli-2 expression 5. SP-CUR + gemcitabine reduced the amount of α-SMA, N-cadherin, and SMO and upregulated hCNT	[135]

α-SMA: alpha-smooth muscle actin; ALT: alanine aminotransferase; AST: aspartate aminotransferase; BLPP: biotin-/lactobionic acid–modified poly(ethylene glycol)-poly(lactic-co-glycolic acid)-poly(ethylene glycol); CK: creatine kinase; CKMB: creatine kinase MB; hCNT: human concentrative nucleoside transporter; H&E: hematoxylin and eosin; LDH: lactate dehydrogenase; NF-қB: nuclear factor-kappa beta; PCNA: proliferating cell nuclear antigen; SHH: sonic hedgehog; SMO: smoothened; VEGF: vascular endothelial growth factor; 5-FU: 5-fluorouracil.
